# Supplementary material for: Resolving the full spectrum of human genome variation using Linked-Reads
Source: Genome Res. 2019 Apr;29(4):635–45. doi: 10.1101/gr.234443.118 (PMC6442396; doi:10.1101/gr.234443.118)
Supplement: Supplemental Material [file supp_gr.234443.118_Supplemental_longranger-2.2.zip › longranger-2.2.tar.bz2!/lr2.2-public/lib/stan/cmdstan/stan/doc/api/html/structstan_1_1math_1_1value__type_3_01std_1_1vector_3_01_t_01_4_01_4.html]

Stan: stan::math::value\_type< std::vector< T > > Struct Template Reference


|  |  |
| --- | --- |
|  | Stan  2.5.0  probability, sampling & optimization |


- Main Page
- Related Pages
- Namespaces
- Classes
- Files

- Class List
- Class Index
- Class Hierarchy
- Class Members

All Classes Namespaces Files Functions Variables Typedefs Enumerations Enumerator Friends Macros Pages

- stan
- math
- value\_type< std::vector< T > >

Public Types |
List of all members

stan::math::value\_type< std::vector< T > > Struct Template Reference

Template metaprogram class to compute the type of values stored in a standard vector.
More...

`#include <value_type.hpp>`

|  |  |
| --- | --- |
| Public Types | |
| typedef std::vector< T >  ::value\_type | type |
|  | Type of value stored in a standard vector with type `T` entries. More... |
|  | |

## Detailed Description

### template<typename T> struct stan::math::value\_type< std::vector< T > >

Template metaprogram class to compute the type of values stored in a standard vector.

Template Parameters
:   |  |  |
    | --- | --- |
    | T | type of elements in standard vector. |

Definition at line 44 of file value\_type.hpp.

## Member Typedef Documentation

template<typename T >

typedef std::vector<T>::value\_type stan::math::value\_type< std::vector< T > >::type

Type of value stored in a standard vector with type `T` entries.

Definition at line 50 of file value\_type.hpp.

---

The documentation for this struct was generated from the following file:

- src/stan/math/meta/value\_type.hpp


---

[ Stan Home Page ]

*© 2011–2014,
Stan Development Team.*
